# Modelling Free-Living and Particle-Associated Bacterial Assemblages across the Deep and Hypoxic Lower St. Lawrence Estuary

**DOI:** 10.1128/mSphere.00364-20

**Published:** 2020-05-20

**Authors:** Ting Ting Cui, Travis J. Dawson, Susan McLatchie, Katherine Dunn, Joseph Bielawski, David A. Walsh

**Affiliations:** a Department of Biology, Concordia University, Montreal, Quebec, Canada; b Department of Biology, Dalhousie University, Halifax, Nova Scotia; University of Wisconsin—Madison

**Keywords:** bacterial diversity, estuary, marine microbiology

## Abstract

The Estuary and Gulf of St. Lawrence (EGSL) in eastern Canada are among the largest and most productive coastal ecosystems in the world. Very little information on bacterial diversity exists, hampering our understanding of the relationships between bacterial community structure and biogeochemical function in the EGSL. During the productive spring period, we investigated free-living and particle-associated bacterial communities across the stratified waters of the Lower St. Lawrence Estuary, including the particle-rich surface and bottom boundary layers. Modelling of community structure based on 16S rRNA gene and transcript diversity identified bacterial assemblages specifically associated with four habitat types defined by water mass (upper water or lower water column) and size fraction (free living or particle associated). Assemblages from the upper waters represent sets of cooccurring bacterial populations that are widely distributed across Lower St. Lawrence Estuary surface waters and likely key contributors to organic matter degradation during the spring. In addition, we provide strong evidence that particles in deep hypoxic waters and the bottom boundary layer support a metabolically active bacterial community that is compositionally distinct from those of surface particles and the free-living communities. Among the distinctive features of the bacterial assemblage associated with lower-water particles was the presence of uncultivated lineages of Deltaproteobacteria, including marine myxobacteria. Overall, these results provide an important ecological framework for further investigations of the biogeochemical contributions of bacterial populations in this important coastal marine ecosystem.

**IMPORTANCE** The Estuary and Gulf of St. Lawrence (EGSL) in eastern Canada is an appealing ecosystem for studying how microbial communities and metabolic processes are related to environmental change. Ocean and climate variability result in large spatiotemporal variations in environmental conditions and oceanographic processes. The EGSL is also exposed to a variety of additional human pressures that threaten its integrity and sustainable use, including shipping, aquiculture, coastal development, and oil exploration. To monitor and perhaps mitigate the impacts of these human activities on the EGSL, a comprehensive understanding of the biological communities is required. In this study, we provide the first comprehensive view of bacterial diversity in the EGSL and describe the distinct bacterial assemblages associated with different environmental habitats. This work therefore provides an important baseline ecological framework for bacterial communities in the EGSL useful for further studies on how these communities may respond to environmental change.

## Abstract (precis)

The Estuary and Gulf of St. Lawrence (EGSL) in eastern Canada is an appealing ecosystem for studying how microbial communities and metabolic processes are related to environmental change. Ocean and climate variability result in large spatiotemporal variations in environmental conditions and oceanographic processes. The EGSL is also exposed to a variety of additional human pressures that threaten its integrity and sustainable use, including shipping, aquiculture, coastal development, and oil exploration. To monitor and perhaps mitigate the impacts of these human activities on the EGSL, a comprehensive understanding of the biological communities is required. In this study, we provide the first comprehensive view of bacterial diversity in the EGSL and describe the distinct bacterial assemblages associated with different environmental habitats. This work therefore provides an important baseline ecological framework for bacterial communities in the EGSL useful for further studies on how these communities may respond to environmental change.

## INTRODUCTION

Bacterial communities are a diverse component of aquatic ecosystems and important drivers of biogeochemical cycles (1, 2). Bacterioplankton populations exhibit habitat preferences that reflect adaptations to environmental factors such as temperature, salinity, and oxygen as well as the composition and concentration of organic and inorganic nutrients, resulting in the large-scale spatiotemporal diversity patterns observed in aquatic ecosystems (3–5). Bacterioplankton also exhibit distributions and adaptations in relation to the small-scale environmental heterogeneity characteristic of aquatic environments (6, 7). Suspended particulate matter, including living and dead planktonic organisms as well as particles formed by the aggregation of extracellular organic and inorganic material, provide microenvironmental complexity to the aquatic environment (8). Carbon and nutrient concentrations are typically higher on these particles than in the surrounding water column, resulting in microniches of concentrated substrates that are colonized by bacteria and are hot spots of microbial activity (8, 9). Bacterial taxa are often characterized as possessing either a particle-associated or a free-living lifestyle, depending on whether they are attached to this suspended material (7). Differences in cell abundance, morphology, and phylogenetic composition as well as metabolic traits and activities have been shown between free-living and particle-associated bacteria (10–12). These particles and their associated bacteria play an important role in the carbon cycle through organic matter remineralization and the transport of carbon and microorganisms from the surface to deep waters and sediments (8, 13, 14).

Particles and their associated bacteria are well studied in the surface waters of lakes and oceans and, to a lesser extent, in the deep ocean. A much more poorly studied particle-rich environment is the bottom boundary layer of marine ecosystems, which is a turbulent region of the water column just above the sea floor (15). Typically, the bottom boundary layer in the coastal ocean has a large amount of particulate organic matter suspended by turbulence, including particles sinking from the surface. Bottom boundary layers in coastal settings are also important because microbial decomposition of organic matter is compressed vertically, making them susceptible to hypoxia (16). Few studies have investigated bacterial diversity or activity in the bottom boundary layer. Owing to the distinct physiochemical characteristics of the bottom boundary layer, it may be colonized by distinctive bacterial taxa important to marine biogeochemistry. This is exemplified by a study of the bottom boundary layer communities along the Oregon continental shelf in the northeast Pacific Ocean, which identified poorly described bacterial lineages, but did not differentiate between free-living and particle-associated fractions (17).

The Estuary and Gulf of St. Lawrence (EGSL) in eastern Canada is among the largest and most productive coastal ecosystems in the world. The diversity and activity of autotrophic phytoplankton communities have been extensively investigated in the EGSL (18). Also, important microbially mediated biogeochemical processes involved in carbon and nutrient cycling have received attention (19–23). Previous studies have reported on the distribution of specific bacterial taxa across the estuarine surface salinity gradient (24) and on the distribution of metabolic pathways across the stratified waters using meta-omics approaches (25). However, no information on bacterial community diversity exists for the EGSL, hampering our understanding of the relationships between bacterial community structure and biogeochemical function in this important marine ecosystem.

A common approach for investigating bacterial community structure and activity is through analysis of 16S rRNA gene (rDNA) and transcript (rRNA) diversity and abundances (26–29). If one assumes that ribosome abundance increases with faster growth and decreases during starvation, as has been shown in bacterial culture, than the 16S rRNA/rDNA ratio may serve as a proxy for growth rate and general levels of metabolic activity. However, correlations between rRNA/rDNA and activity are not always consistent, can differ between taxonomic groups, and may be influenced by ecological strategies (30). Hence, the rRNA/DNA ratio should be interpreted with caution, and a recent critical evaluation suggested the rRNA/rDNA ratio conservatively represents the protein synthesis potential (PSP) of a population (31).

In this study, we investigated free-living and particle-associated bacterial community structure and activity in the Lower St. Lawrence Estuary (LSLE). The LSLE is deep and strongly stratified and possesses a characteristic cold intermediate layer and bottom waters that are persistently hypoxic (32). The LSLE is a major deposition zone for suspended particulate matter, and the bottom boundary layer is well developed, particularly at the head of the LSLE, where increased turbidity is caused by friction of the tides at the sediment-water interface (33). Our main objective was to determine the habitat preferences for bacterial populations residing in the LSLE. We tested the specific hypothesis that the particles in the deep hypoxic water of the LSLE support a distinct bacterial community compared to that of surface particles and the free-living communities. 16S rRNA amplicon data sets from the DNA and RNA fractions were generated from two size fractions representing the free-living (0.22 to 2.7 μm) and particle-associated (>2.7 μm) bacterial communities. We used multivariate approaches to explore bacterial community structure across the LSLE and showed that samples could be classified to one of four discrete habitat types defined by depth and size fraction. Next, we used a Bayesian modelling approach to identify and describe bacterial assemblages specifically associated with each habitat type. Finally, we used the PSP values of the dominant members of the bacterial assemblages to assess their potential contributions to the metabolic activities occurring in each of the defined habitat types.

## RESULTS AND DISCUSSION

### Environmental setting.

**FIG 1:**
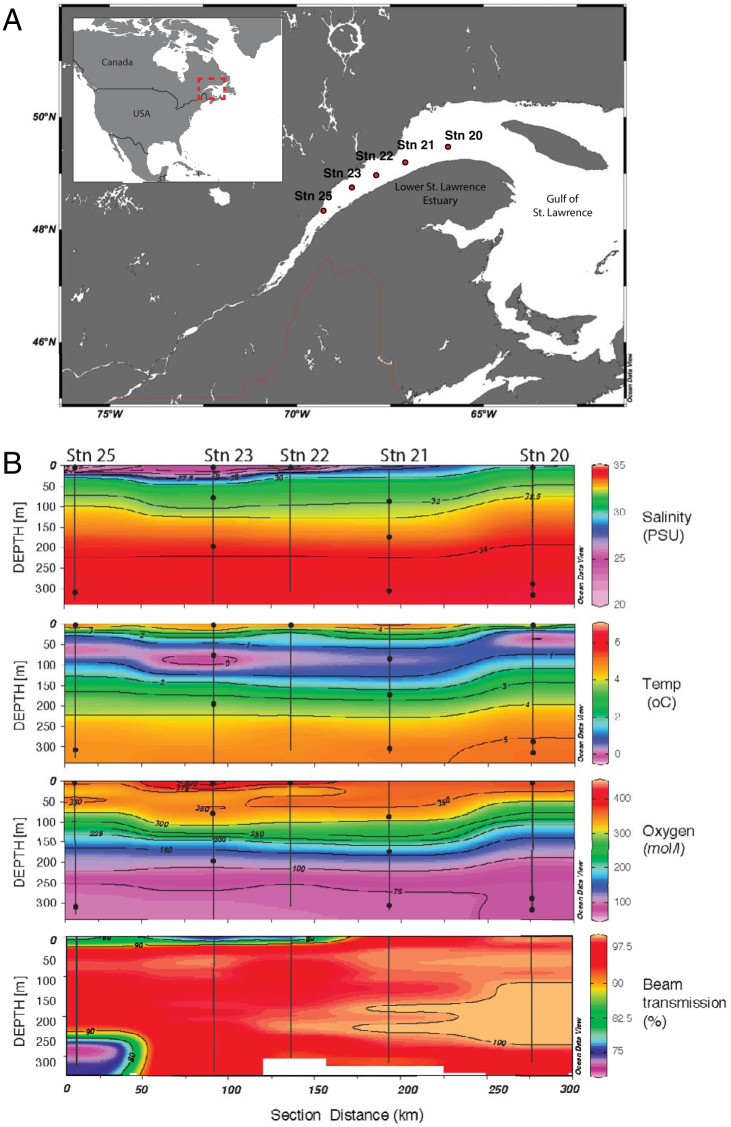
Sampling locations (A) and environmental profiles (B) of the LSLE during the sampling campaign from 16 to 21 May 2011. Maps and plots were generated with Ocean Data Viewer (65).

We investigated bacterial communities along a five-station (S25 to S20) transect of the LSLE in May 2011 (Fig. 1A). The surface salinity gradient ranged from 21.2 to 28.7 practical salinity units (PSU) with a slight depression in the central region (S22) (Fig. 1B; Table 1) due to freshwater input from the Saguenay River. Highly stratified and distinct water masses were apparent. The upper water column (1 to 120 m) was characterized by a vertical salinity gradient due to estuarine circulation and the cold intermediate layer. Below the cold intermediate layer, the lower water column was characterized by higher-salinity water and a vertical oxygen gradient. Particle-rich environments were present in the upper and lower water columns. Turbidity (i.e., beam transmission) and measurements of suspended particulate matter showed a general decrease in particle abundance along the surface salinity gradient (Fig. 1B; Table 1). A similar gradient was observed in the bottom layer, and the greatest particle density occurred in the well-developed bottom boundary layer at S25 (21.35 mg/liter of suspended particulate material).

**TABLE 1:** Environmental parameters for samples collected along the Lower St. Lawrence Estuary transect from 16 to 21 May 2011

Station	Depth (m)	Temp (°C)	Salinity (PSU)	Oxygen (μmol /kg)	Fluorescence	Beam transmission (%)	SPM (mg/liter)^*a*^
25	3	5.49	21.20	369.73	0.28	78	3.46
25	325	4.71	34.36	66.10	0.06	74	21.35
23	3	4.74	24.25	412.61	12.31	69	5.80
23	80	−0.17	31.78	331.49	0.21	99	4.48
23	299	3.66	33.87	104.92	0.12	99	2.64
22	3	5.43	22.16	416.06	0.85	73	9.82
21	80	0.47	31.81	336.80	0.07	100	2.65
21	180	2.49	33.36	166.34	0.01	100	
21	314	4.98	34.50	69.65	0.01	92	6.27
20	3	6.16	28.67	371.80	0.01	100	2.67
20	280	5.03	34.52	77.06	0.01	99	
20	316	5.10	34.56	79.23	0.01	97	3.35

a SPM, solid particulate material.

### Bacterial diversity and community structure.

**FIG 2:**
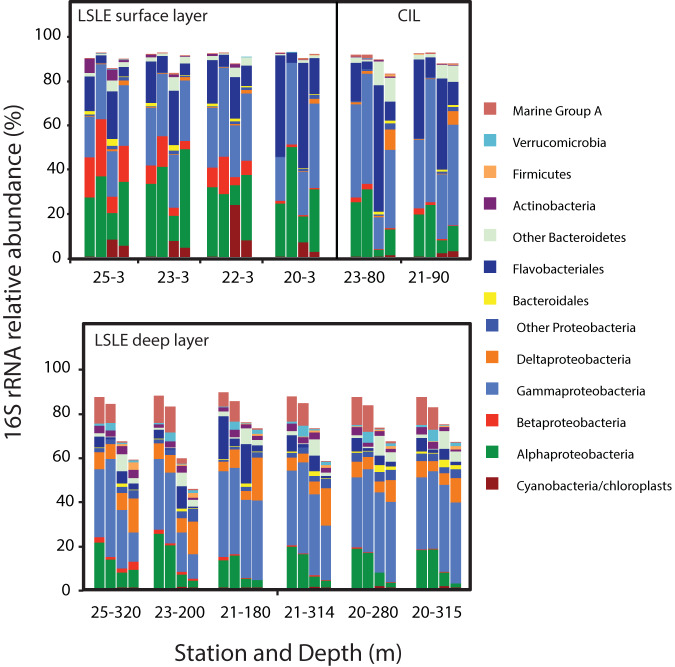
Taxonomic compositions of LSLE 16S rRNA amplicon data sets from the free-living (FL) and particle-associated (PA) size fractions. Columns are arranged in the following order for each sample location: 1, FL-rDNA; 2, FL-rRNA; 3, PA-rDNA; 4, PA-rRNA fractions.

Surface waters were dominated by typical marine lineages within the *Alphaproteobacteria*, *Gammaproteobacteria*, and *Flavobacteriales*, although typically, freshwater lineages within the *Betaproteobacteria* and *Actinobacteria* were also common in the upper region of the LSLE (Fig. 2). Cyanobacteria and chloroplasts were abundant (>20%) in the particle-associated (PA) fraction from station 22, revealing potential bloom conditions in this region. The lower water column was differentiated from the upper water column communities by the presence of Deltaproteobacteria and the marine group A clade.

**FIG 3:**
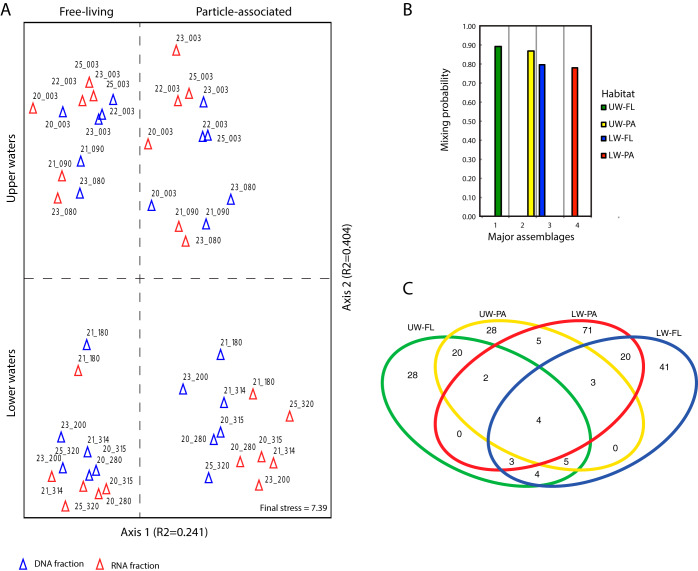
Habitat partitioning of LSLE samples by depth and size fraction. (A) NMS ordination analysis of 16S rRNA OTUs. Samples are color coded by rDNA or rRNA fractions. PC-ORD reports the stress value as Kruskal’s stress formula 1 multiplied by 100. (B) Mixing probabilities of the major assemblages of OTUs identified by BioMiCo analysis. (C) Venn diagram of the distribution of principal OTUs (posterior probability [PP] > 0.002) within the four dominant assemblages.

Variability in bacterial community structure was assessed using unconstrained nonmetric multidimensional scaling (NMS) analysis of bacterial operational taxonomic unit (OTU) distributions across the rDNA and rRNA data sets. In the NMS ordination plot, samples from the upper water column were well separated from lower water column samples along axis 2 (Fig. 3A). Samples from the free-living fractions were separated from the particle-associated fractions along axis 1 (Fig. 3A). Based on these results, at the time of sampling, it appeared that the LSLE bacterial samples originated from four dissimilar habitat types defined by depth and size fraction: upper-water free-living (UW-FL), upper-water particle-associated (UW-PA), lower-water free-living (LW-FL), and lower-water particle-associated (LW-PA) habitats. The DNA and RNA fractions both exhibited this pattern, that is, samples were grouped in the ordination based on habitat compartment and not by nucleic acid type (Fig. 3A). This observation was verified by analysis of similarity (ANOSIM), which demonstrated a greater difference between particle-associated and free-living fractions (UW, *R* = 0.987, *P* = 0.001; LW, *R* = 0.583, *P* = 0.001) than between rDNA and rRNA fractions (UW, *R* = 0.147, *P* = 0.034; LW, *R* = 0.13, *P* = 0.054). These results reveal similar and overlapping distributions of OTUs in the rDNA and rRNA data sets.

### Habitat-associated bacterial assemblages.

To characterize bacterial diversity and distributions across the four habitat types, we assessed the strength of OTU associations with each habitat using a hierarchical community structure modelling approach termed BioMiCo (34). Given a set of categorized samples, BioMiCo will identify assemblages (i.e., mixtures of OTUs with a tendency to cooccur) associated with each category. In a previous study, BioMiCo was used to identify and describe bacterial assemblages associated with seasonal periods in the coastal ocean (35). For the LSLE, we set the categories as the four habitat types revealed by the NMS analysis (UW-FL, UW-PA, LW-FL, and LW-PA). A single major assemblage contributing between 78% and 89% of the posterior density to each category was identified (Fig. 3B). This result demonstrates that the four habitat types were strongly differentiated from each other by the existence of habit-specific bacterial assemblages.

Owing to the probabilistic modelling employed in BioMiCo, OTUs are not necessarily restricted to a single assemblage but can be associated with multiple assemblages with various strengths. The OTU association strengths across assemblages can provide insight into physicochemical and biological factors shaping bacterial community structure and activity within the LSLE. For example, we identified a total of 234 OTUs that exhibited a strong association (posterior probability of >0.002) with at least one of the four habitat assemblages (Fig. 3C). We refer to these as the principal OTUs of an assemblage, similarly other studies (35). Principal OTUs contained between 26% and 74% of 16S rDNA or rRNA sequences for each sample and so captured a significant amount of the sequence data, including some of the most abundant OTUs. The majority (168) of the principal OTUs were strongly associated with a single assemblage, reflecting bacterial populations with a relatively constrained distribution defined by depth interval (UW or LW) and size class (FL or PA) (Fig. 3C). The other 66 principal OTUs were associated with two or more habitat assemblages, suggesting a less constrained distribution and perhaps a more generalist lifestyle for these bacterial populations. The greatest overlap of principal OTUs was observed between the free-living and particle-associated assemblages from the same depth interval (20 shared principal OTUs in both cases).

**FIG 4:**
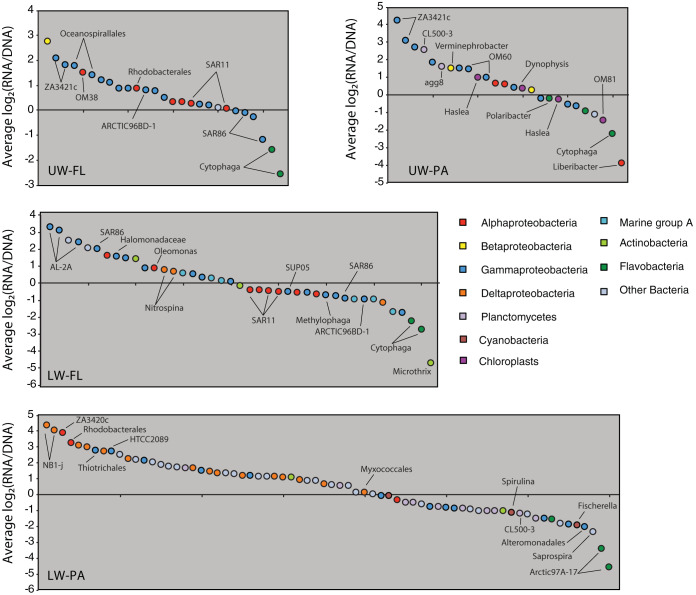
The protein synthesis potential (PSP) values as proxies for metabolic activity of the principal OTUs unique to a single habitat category. PSP values were calculated as log_2_(rRNA/rDNA) for each OTU. Each panel represents one of the four habitat types: UW-FL, upper water column, free living; UW-PA, upper water column, particle associated; LW-FL, lower water column, free living; LW-PA, lower water column, particle associated. Each OTU is colored based on its high-level taxonomic identity. Some OTUs have been labeled with a more specific taxonomic identity. The full taxonomic data are available in Tables S1, S2, S4, and S5 in the supplemental material.

The BioMiCo analysis revealed that bacterial OTUs exhibited strong associations with a habitat type in the LSLE. In the following section, we used the OTU distributions among the four major assemblages to describe habitat preferences and lifestyle strategies of bacterial populations in the LSLE. We also used the PSP values, calculated as log_2_(RNA/DNA) ratios of the principal OTUs, as a proxy for growth rate and general metabolic activity based on the assumption that ribosome abundance increases with growth rate (26–29). A wide range in PSP values was observed among principle OTUs in all assemblages (Fig. 4).

10.1128/mSphere.00364-20.1 TABLE S1 Relative abundance and taxonomic identity of dominant OTUs (PP > 0.002) from the upper water column free-living assemblage. Values in parentheses adjacent to sample identifiers correspond to the percentage of reads that correspond to 10 reads per sample. The highlighted values are those that correspond to ≥10 reads. Only samples where an OTU was represented by ≥10 sequences in both the DNA and RNA fractions were included in the PSP calculation to minimize biases associated with low-abundance OTUs. Download Table S1, XLSX file, 0.1 MB. Copyright © 2020 Cui et al. 2020 Cui et al. This content is distributed under the terms of the Creative Commons Attribution 4.0 International license.

10.1128/mSphere.00364-20.2 TABLE S2 Relative abundance and taxonomic identity of dominant OTUs (PP > 0.002) from the upper water column particle-associated assemblage. Values in parentheses adjacent to sample identifiers correspond to the percentage of reads that correspond to 10 reads per sample. The highlighted values are those that correspond to ≥10 reads. Only samples where an OTU was represented by ≥10 sequences in both the DNA and RNA fractions were included in the PSP calculation to minimize biases associated with low-abundance OTUs. Download Table S2, XLSX file, 0.1 MB. Copyright © 2020 Cui et al. 2020 Cui et al. This content is distributed under the terms of the Creative Commons Attribution 4.0 International license.

### Bacterial assemblages associated with LSLE surface waters.

Freshwater and marine ecosystems are inhabited by phylogenetically distinct bacterial communities (36), making estuarine salinity gradients sites of rapid species turnover. Despite the salinity variations in LSLE surface waters (21 to 29 PSU), size fraction (free living versus particle associated) appeared to be the principal factor in shaping bacterial community composition and not location along the salinity gradient. Previous work has shown that massive bacterial cell death occurs in the Upper St. Lawrence Estuary (37, 38); hence, the most intense turnover between freshwater and marine communities occurred upstream of the LSLE. Therefore, the majority of primary OTUs within the UW-FL and UW-PA assemblages likely represent taxa indigenous to the brackish-marine LSLE surface waters rather than allochthonous taxa introduced with freshwater. In support of this, the zones of most rapid taxonomic turnover in other systems such as the Baltic Sea and the Delaware Bay estuary occur at salinity concentrations around 10 to 20 PSU, which occurs upstream of S25 (28, 39).

The UW-FL and UW-PA assemblages each comprised 28 unique principle OTUs (Fig. 3C). As these two assemblages represent sets of cooccurring bacterial populations that are widely distributed across LSLE surface waters, we hypothesize that they are key contributors to organic matter degradation in this marine ecosystem during spring. Both assemblages contained OTUs assigned to taxa typically associated with productive marine environments, including *Alphaproteobacteria* (*Rhodobacterales*, OM38, and SAR11), *Gammaproteobacteria* (*Oceanospirillales*, ZA3412c, and SAR86), and *Flavobacteria* (*Cytophaga*, *Polaribacter*) (40) (Fig. 4; see also Tables S1 and S2 in the supplemental material). An assemblage of a similar taxonomic composition was recently described from the spring bloom in the North Atlantic Ocean (35). Interestingly, PSP values varied among the OTUs, suggesting differences in their relative metabolic activities. For both the FL and PA assemblages, the most positive PSP values were reported for OTUs assigned to *Gammaproteobacteria* (ZA4311c), while the most negative were reported for OTUs assigned to *Flavobacteria* (*Cytophaga*) (Fig. 4). Previous temporal studies of bacterial succession across phytoplankton blooms have shown *Flavobacteria* tend to be most active early, while *Gammaproteobacteria* become more active at later stages of the bloom (41). Hence, the differences in PSP values here suggest the assemblages are associated with the later stages of the spring bloom in the LSLE.

**FIG 5:**
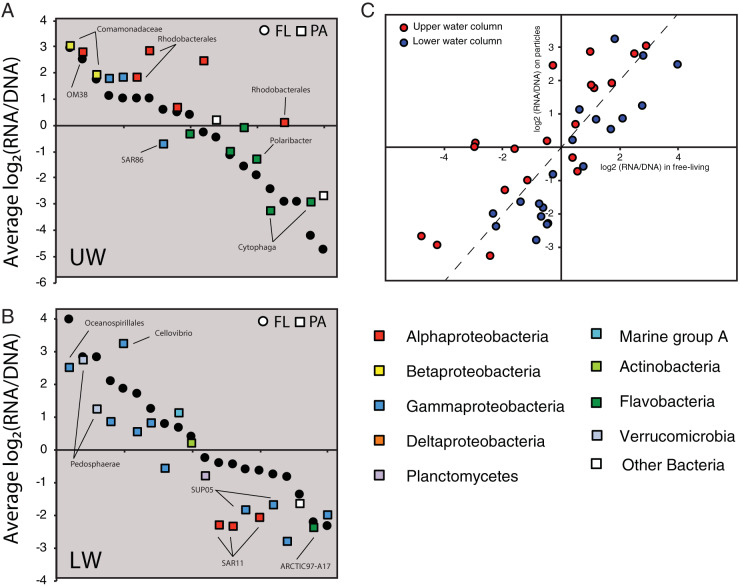
The PSP values as proxies for metabolic activity of the principal OTUs shared between the free-living and particle-associated assemblages from either the UW (A) or LW (B) column of the LSLE. Each OTU is colored based on its high-level taxonomic identity. Some OTUs are labeled with a more specific taxonomic identity. The full taxonomic data are available in Tables S3 and S6. (C) A scatterplot of the PSP values against each other for the shared principal OTUs in the assemblages from the upper or lower water column.

Although unique assemblages were associated with the upper-water FL and PA communities, approximately 40% (20) of principal OTUs in each assemblage were common exclusively to both UW assemblages (Fig. 5A; see also Table S3). This overlap suggests relatively high connectivity between surface free-living and particle-associated communities and agrees with other studies that have shown that the main differences between free-living and particle-associated communities is in the relative abundance of shared members rather than the complete partitioning between habitats (42). Hence, there appears to be considerable exchange between free-living and particle-associated communities in the upper water column as populations colonize and detach from particles, as has been predicted from other coastal communities (10).

10.1128/mSphere.00364-20.3 TABLE S3 Relative abundance and taxonomic identity of dominant OTUs (PP > 0.002) shared between the upper water column free-living and particle-associated assemblages. Values in parentheses adjacent to sample identifiers correspond to the percentage of reads that correspond to 10 reads per sample. The highlighted values are those that correspond to ≥10 reads. Only samples where an OTU was represented by ≥10 sequences in both the DNA and RNA fractions were included in the PSP calculation to minimize biases associated with low-abundance OTUs. Download Table S3, XLSX file, 0.1 MB. Copyright © 2020 Cui et al. 2020 Cui et al. This content is distributed under the terms of the Creative Commons Attribution 4.0 International license.

The PSP values for the shared UW principal OTUs provided some additional insight into population dynamics and activity. For example, we observed a positive relationship when we plotted the PSP values for each habitat type against each other (*R*^2^ = 0.69) (Fig. 5C), demonstrating that the most active principal OTUs in the particle-associated fraction were also typically the most active in the free-living fraction. However, the majority of the shared OTUs exhibited higher PSP values on particles than when in the free-living fraction. These results suggest that although these shared OTUs are abundant in the free-living fraction, they show a higher per cell level of activity, and perhaps elevated growth rate, when associated with particles.

Several taxonomic features differentiated the two surface assemblages. For example, the UW-PA assemblage included principle OTUs assigned to chloroplasts (*Dinophysis*, *Haslea*, and OM81). *Cyanobacteria* and chloroplasts were common components of the surface particle-associated 16S rDNA and rRNA data sets in general but were consistently less abundant in the rRNA faction than in the rDNA fraction for paired data sets (Fig. 2). This observation suggests the chloroplast OTUs originate from less active or decaying phytoplankton. Although, short-term temporal variability in phytoplankton gene expression patterns could confound our PSP values due to the diel growth cycle of phytoplankton (43). Principle OTUs assigned to *Planctomycetes* (CL500-3 and agg8) were also specifically associated with the UW-PA assemblage and exhibited positive PSP values (Fig. 4). *Planctomycetes* are often implicated in the degradation of algal-derived polysaccharides (44) and proteins (45) and are likely playing an important role in particulate organic matter hydrolysis in the LSLE. A principal OTU assigned to *Actinobacteria* (ACK-M1) was associated with the UW-FL assemblage (Table S1). *Actinobacteria* are typically associated with freshwater and terrestrial ecosystems (46), although marine lineages do exist (47, 48). An additional *Actinobacteria* (Microthrix) principal OTU was also identified in the LW-FL assemblage (Fig. 4). Hence, free-living populations of *Actinobacteria* appear to be widespread in the LSLE water column. Whether the specific *Actinobacteria* detected here represent true marine bacteria or terrestrial/freshwater lineages able to persist in the marine environment remains to be determined.

### Bacterial assemblages associated with LSLE deep waters.

The habitat assemblages from the LSLE lower waters comprised a greater number of principal OTUs than the surface assemblages. There were 41 and 71 principal OTUs unique to the LW-FL and LW-PA assemblages, respectively (Fig. 3C). Compared to that of surface assemblages which exhibited considerable overlap in the taxonomic identity of principle OTUs, we observed a higher degree of taxonomic dissimilarity between the LW-FL and LW-PA assemblages (Fig. 4; see also Tables S4 and S5). This dissimilarity was particularly strong between principal OTUs with positive PSP values. Principal OTUs assigned to *Alphaproteobacteria* and *Gammaproteobacteria* were among those with positive PSP values in both the LW-FL and LW-PA assemblages. However, a distinctive feature of the LW-PA assemblage was the occurrence of principal OTUs assigned to the Deltaproteobacteria (Fig. 4). *Planctomycetes* (CL500-3) were also a distinct feature of the LW-PA assemblage, but were among the OTUs with negative PSP values. The distinct and diverse taxonomic composition of the LW-PA assemblage demonstrated that lower-water particle-associated communities are the most diverse and distinct in the LSLE.

10.1128/mSphere.00364-20.4 TABLE S4 Relative abundance and taxonomic identity of dominant OTUs (PP > 0.002) from the lower water column free-living assemblage. Values in parentheses adjacent to sample identifiers correspond to the percentage of reads that correspond to 10 reads per sample. The highlighted values are those that correspond to ≥10 reads. Only samples where an OTU was represented by ≥10 sequences in both the DNA and RNA fractions were included in the PSP calculation to minimize biases associated with low-abundance OTUs. Download Table S4, XLSX file, 0.1 MB. Copyright © 2020 Cui et al. 2020 Cui et al. This content is distributed under the terms of the Creative Commons Attribution 4.0 International license.

10.1128/mSphere.00364-20.5 TABLE S5 Relative abundance and taxonomic identity of dominant OTUs (PP > 0.002) from the lower water column particle-associated assemblage. Values in parentheses adjacent to sample identifiers correspond to the percentage of reads that correspond to 10 reads per sample. The highlighted values are those that correspond to ≥10 reads. Only samples where an OTU was represented by ≥10 sequences in both the DNA and RNA fractions were included in the PSP calculation to minimize biases associated with low-abundance OTUs. Download Table S5, XLSX file, 0.1 MB. Copyright © 2020 Cui et al. 2020 Cui et al. This content is distributed under the terms of the Creative Commons Attribution 4.0 International license.

10.1128/mSphere.00364-20.6 TABLE S6 Relative abundance and taxonomic identity of dominant OTUs (PP > 0.002) shared between the lower water column free-living and particle-associated assemblages. Values in parentheses adjacent to sample identifiers correspond to the percentage of reads that correspond to 10 reads per sample. The highlighted values are those that correspond to ≥10 reads. Only samples where an OTU was represented by ≥10 sequences in both the DNA and RNA fractions were included in the PSP calculation to minimize biases associated with low-abundance OTUs. Download Table S6, XLSX file, 0.1 MB. Copyright © 2020 Cui et al. 2020 Cui et al. This content is distributed under the terms of the Creative Commons Attribution 4.0 International license.

In addition to the distinctive composition of the LW-PA assemblage, a lower level of connectivity between free-living and particle-associated communities in the lower waters of the LSLE than for the upper waters was also evident. For example, there was a lower contribution of shared principal OTUs exclusive to the lower water column assemblages (20 shared, which represents 32% and 22% for LW-FL and LW-PA assemblages, respectively) (Fig. 5B; see also Table S6). A positive relationship between PSP values in FL and PA assemblages for OTUs was observed (*R*^2^ = 0.78), similar to that for the UW assemblages. However, whereas the shared UW principal OTUs exhibited higher relative activity when associated with particles, the opposite was true for these shared LW principal OTUs. Eighteen of the 20 OTUs shared between LW assemblages exhibited lower PSP values when associated with particles than with the free-living fraction. These results suggest that although these populations are common in the free-living and particle-associated communities, they are metabolically more active while freely suspended in the water column and may be maladapted to conditions, or even dead, on particles.

Particles formed in the surface water sink into the deep ocean, and it was recently shown that bacterial taxa attached to sinking particles can influence the structure of deeper bacterial communities (13). Such a process appears to play a role in structuring the lower water column particle-associated communities in the LSLE. For example, we identified five principal OTUs shared between the UW-PA and LW-PA assemblages. OTUs assigned to *Cyanobacteria* (*Spirulina* and *Fischerella*) were also in the LW-PA assemblage and had negative PSP values (Fig. 4). Moreover, 16S rRNA gene sequences assigned to UW-PA principal OTUs were common in deeper-water particle-associated samples (although not as principal OTUs of the LW-PA assemblage). In contrast, far fewer sequences assigned to LW-PA principal OTUs were identified in upper-water particle-associated samples. We interpret this as support for the notion that sinking particles are transporting surface taxa to the deep water of the LSLE, similarly to observations in the open ocean. However, whether or not these surface populations represent a supply of particulate organic matter for the bottom water community (presumably true for the *Cyanobacteria* and chloroplasts) or remain active in particle degradation remains to be determined.

**FIG 6:**
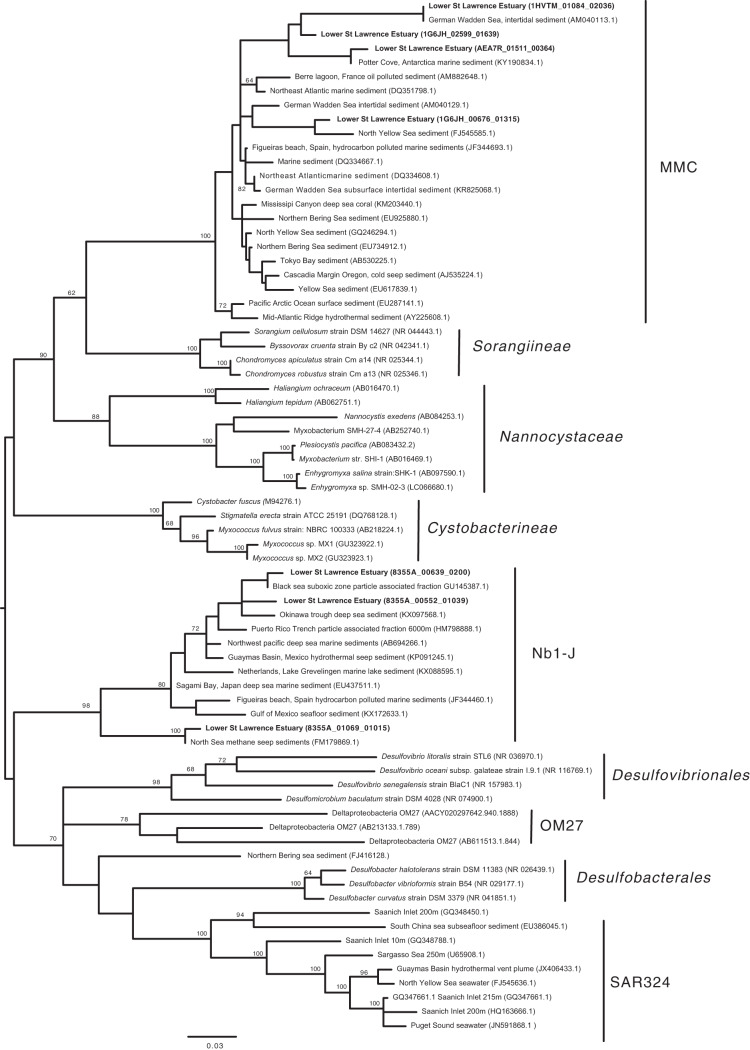
Maximum likelihood phylogenetic analysis of selected 16S rRNA OTUs associated with Deltaproteobacteria. Taxa in bold font are those from the LSLE. The tree was inferred from a MUSCLE alignment (66) comprising the publicly available near-full-length 16S rRNA gene and the short (100 bp) amplicons generated in the present study using MEGA version 6.0 (67).

The particle-rich bottom waters of the LSLE are persistently hypoxic (32). Bacterial respiration in particles may be capable of depleting oxygen concentrations further, creating anoxic microenvironments in particles (16, 49). This process may select for strict or facultative anaerobes (10). The most distinctive feature of the LW-PA assemblage was the occurrence of OTUs assigned to Deltaproteobacteria, which also showed positive PSP values characteristic of an elevated metabolic activity compared to that of other assemblage members. Phylogenetic analysis of these OTUs showed some are members of the uncultivated marine myxobacteria clade (MMC) and the uncultivated Nb1-j clade of Deltaproteobacteria (Fig. 6). The LSLE Nb1-j OTUs were closely related to sequences identified in the suboxic zone of the Black Sea (50), deep sea particles from the Puerto Rican Trench (51), and a range of marine sediments (52, 53). Myxobacteria are also common in marine sediments and have been suggested to be facultative anaerobes (54). These observations further support the notion that the deep LSLE particles represent a distinct bacterial habitat compared to that in the surrounding water column. The metabolic capabilities of these particle-associated communities and the roles they play in LSLE biogeochemistry and the formation of hypoxia warrant further attention.

### Conclusion.

In conclusion, we have identified and described bacterial assemblages associated with contrasting particle-rich environments in the upper and lower waters of the LSLE. We have also provided strong evidence that the particles in the deep hypoxic waters of the LSLE support a distinct bacterial community compared to that of surface particles and the FL communities. The partitioning of samples into four habitats (UW-FL, UW-PA, LW-FL, and LW-PA) may be relatively coarse for a dynamic coastal system defined by gradients in temperature, salinity, nutrient concentration, organic matter composition, and oxygen. Yet, the community structure modelling identified a single assemblage of bacterial taxa strongly associated with each habitat type, supporting the ecological relevance of the habitat designations. These results therefore provide an important ecological framework for further investigations of the biogeochemical contributions of bacterial populations in this important marine ecosystem.

Similarly to other coastal systems (55, 56) and estuaries (11), the phylogenetic compositions differed between FL and PA communities, a pattern that was most pronounced in the deeper waters. In addition, the high PSP values for OTUs strongly associated with the bottom boundary layer support other reports that the bottom boundary layer of coastal systems may be a biogeochemically unique habitat in the ocean (17). Future comparative studies coupling metagenomics and gene expression profiling of the deeper water communities will provide insights into the genomic composition and metabolic capacity of the poorly characterized particle-associated assemblages. The bottom boundary layer is partially composed of suspended particulate matter from surface sediments. It will also be informative to investigate the connectivity between bottom boundary layer and sediment communities to more strictly assess the habitat origin, ecology, and metabolism of the deeper water taxa, such as the Deltaproteobacteria lineages.

## MATERIALS AND METHODS

### Sampling and environmental data.

Seawater samples were collected from five stations (S25, S23, S22, S21, and S20) along a transect of the LSLE using a conductivity, temperature, and depth (CTD) rosette in May 2011. Water samples (2 liters) for DNA and RNA were collected and processed by serially filtering through a Whatman GF/D filter (2.7-μm cutoff) and a 0.22-μm Sterivex-GV filter via a peristaltic pump. Filters were either stored in 1.8 ml of a sucrose lysis buffer (40 mM EDTA [pH 8.0], 50 mM Tris [pH 8.3], 0.75 M sucrose) for DNA extraction or RNAlater for RNA extraction. Environmental data collection and analysis were reported by Colatriano et al. (25).

### Nucleic acid extraction.

DNA extraction was performed from 0.22-μm Sterivex filters for free-living bacteria and from 2.7-μm GF/D filters for particle-associated bacteria using a modified protocol described by Zaikova et al. (57). Cell lysis and digestion were conducted by thawing and adding 100 μl of lysozyme (concentration [conc.] 125 mg in 1,000 μl) and 20 μl of RNase A (conc. 10 μg/ml) to the filters containing biomass (either directly into the Sterivex filter or added to the GF/D filter in a 50-ml Falcon tube) followed by incubation in a rotating oven at 37°C for 1 h. We then added 100 μl of proteinase K (conc. 10 mg/ml) and 100 μl of 20% SDS to the filter and rotated again for 2 h more in the hybridization oven at 55°C. Lysate was transferred into 2-ml centrifuge tubes; 0.583 volumes of MPC protein precipitation solution (MPC) from MasterPure from Epicenter was added in order to precipitate the protein. A protein pellet was formed after centrifuging at a speed >10,000 × *g* at 4°C for 10 min. The supernatant was transferred into another tube, and 0.95 volumes of isopropanol was added to precipitate the DNA, which was washed twice with 750 μl of 70% ethanol. Ethanol was removed, and the pellet was resuspended with 25 μl Tris buffer [pH 7.5 to 8.0].

RNA was extracted from separate filters than those used for the DNA using the mirVana miRNA isolation kit (Life Technologies, Burlington, ON, Canada) and the RNeasy RNA cleanup kit (Qiagen) according to the modified methodology described by Stewart et al. (58). Samples were thawed, and the RNAlater (Invitrogen) was removed. mirVana lysis buffer was added to the Sterivex filter unit or the GF/D filter and vortexed to lyse bacterial cells attached to the filter. Total RNA was then extracted from the lysate according to the mirVana protocol. Extracted RNA was treated with 2 units of DNase (New England BioLabs) at 65°C for 1 to 2 h to remove DNA and concentrated using the RNeasy RNA cleanup kit (Qiagen). The RNA extracted from samples was PCR amplified using the protocol described below and run on a 1% agarose gel in order to detect any DNA contamination after DNase treatment, in which case, the DNase treatment and RNA cleanup were repeated on those samples.

### Generation of 16S rRNA sequence data from environmental DNA and RNA.

cDNA synthesis from 1 ng of RNA was performed by reverse transcription (RT) reactions using M-MLV reverse transcriptase (Invitrogen) and reverse primer DW926R. RT reactions proceeded by heat denaturing RNA at 65°C for 5 min and subsequently incubating at 37°C for 50 min. For PCR from genomic DNA or cDNA, we used universal primers for the V5 region of the 16S rRNA gene (DW786F, 5′-GATTAGATACCCTSGTAG-3′, and DW926R, 5′-CCGTCAATTCMTTTRAGT-3′) that were modified from those used by Baker et al. (59) to eliminate bias against marine *Alphaproteobacteria*. PCR mixtures (50 μl total volume) contained 0.5 μM each primer, 1× Phire reaction buffer containing 1.5 mM MgCl_2_ (Finnzymes, Thermo Fisher Scientific), 0.2 mM deoxynucleoside triphosphates (dNTPs), and 1 U of Phire Hot Start II DNA polymerase (Finnzymes, Thermo Fisher Scientific). Cycling conditions involved an initial 3-min cell lysis/denaturing step, followed by 30 cycles of 5 s at 98°C, 5 s at 49°C, and 10 s at 72°C, and a final elongation step of 1 min at 72°C. Sample barcoding, library construction, and DNA sequencing were performed using an Ion Torrent PGM system on a 316 chip with the Ion Sequencing 200 kit as described in reference 60.

### Bioinformatic analysis of 16S rRNA gene sequences.

V5 16S rRNA sequences were analyzed using the Mothur pipeline (61). Sequences with an average quality of <17, length of <100 bp, or that did not match the IonXpress barcode and both the PCR forward and reverse primer sequences were discarded. Potential chimeric sequences were identified using UCHIME (62) and also discarded. Sequences were clustered into operational taxonomic units (OTUs) at 97% identity using the furthest-neighbor algorithm. Sequences and OTUs were assigned to taxonomic groups using the Silva database (63) and the Greengenes taxonomy (64) using the Wang method and a bootstrap value cutoff of >60% as implemented in Mothur. We included OTUs assigned to *Cyanobacteria* and chloroplasts in further analyses. In doing so, we may have biased our estimates of other bacterial taxa, but the *Cyanobacteria* and chloroplast data provided valuable information on phytoplankton dynamics in surface waters and transport of particles from surface to deeper waters.

### Statistical analyses.

Nonmetric multidimensional scale (NMS) ordination of OTU relative abundances was performed using Bray-Curtis distances forced to 2 axes with 250 iterations and a stability criterion of 10^−4^, implemented in PC-ORD. ANOSIM was used to compare intra- versus intersample similarity indices. PSP values for principal OTUs were calculated as the average of log_2_(rRNA/rDNA) of paired samples. Only samples where an OTU was represented by ≥10 sequences in both the DNA and RNA fractions were included in the PSP calculation to minimize biases associated with low-abundance OTUs.

The Bayesian modelling framework called BioMiCo was used to infer how assemblages of OTUs are mixed to form communities. A complete description of the model was provided by Shafiei et al. (34). The model was supplied with 16S rRNA OTU abundance data, and the community composition of each sample was modeled by applying a Dirichlet prior to the parameters of the multinomial distribution. Under BioMiCo, the inference of community structure employs supervised learning. Collapsed Gibbs sampling (2,000 iterations) was used to learn the posterior distribution of the mixture weights given the assigned habitat types (surface free living, surface particle associated, deep free living, deep particle associated) for the 48 samples collected from the LSLE transect. It is during this “training phase” that the model learns which assemblages are associated with the habitat types. A large posterior mixing probability is taken as evidence of an association between an assemblage and a habitat type. To guard against unnecessary community complexity, we choose the concentration parameter values for the Dirichlet prior to be close to zero (α_θ_ = 0.01). Because we had no prior preference for a particular assemblage, we used a symmetric Dirichlet prior. The sparsity introduced by this prior solves model identifiability issues, reduces model variance, and improves model interpretability (34). Indeed, using the prior in this way was effective for these data. The model was run by assuming 100 possible assemblages. The same strategy was employed with the prior placed on the OTU composition of an assemblage; i.e., we employed a sparse symmetric Dirichlet prior with concentration parameters set close to zero (α_ϕ_ = 0.01).

### Data availability.

All 16S rRNA sequence data are available as a Dryad data set at https://doi.org/10.5061/dryad.4f4qrfj8d.

## References

[1] Kujawinski EB 2011 The impact of microbial metabolism on marine dissolved organic matter. Annu Rev Mar Sci 3:567–599. doi:10.1146/annurev-marine-120308-081003. 21329217

[2] Penn JL, Weber T, Chang BX, Deutsch C 2019 Microbial ecosystem dynamics drive fluctuating nitrogen loss in marine anoxic zones. Proc Natl Acad Sci U S A 116:7220–7225. doi:10.1073/pnas.1818014116. 30910952 PMC6462081

[3] Wright JJ, Konwar KM, Hallam SJ 2012 Microbial ecology of expanding oxygen minimum zones. Nat Rev Microbiol 10:381–394. doi:10.1038/nrmicro2778. 22580367

[4] Sunagawa S, Coelho LP, Chaffron S, Kultima JR, Labadie K, Salazar G, Djahanschiri B, Zeller G, Mende DR, Alberti A, Cornejo-Castillo FM, Costea PI, Cruaud C, d’Ovidio F, Engelen S, Ferrera I, Gasol JM, Guidi L, Hildebrand F, Kokoszka F, Lepoivre C, Lima-Mendez G, Poulain J, Poulos BT, Royo-Llonch M, Sarmento H, Vieira-Silva S, Dimier C, Picheral M, Searson S, Kandels-Lewis S, Tara Oceans coordinators, de Vargas C, Gorsky G, Grimsley N, Hingamp P, Iudicone D, Jaillon O, Not F, Ogata H, Pesant S, Speich S, Stemmann L, Sullivan MB, Weissenbach J, Wincker P, Karsenti E, Raes J, Acinas SG, Bork P, 2015 Structure and function of the global ocean microbiome. Science 348:1261359. doi:10.1126/science.1261359. 25999513

[5] Chow C-E, Sachdeva R, Cram JA, Steele JA, Needham DM, Patel A, Parada AE, Fuhrman JA 2013 Temporal variability and coherence of euphotic zone bacterial communities over a decade in the Southern California Bight. ISME J 7:2259–2215. doi:10.1038/ismej.2013.122. 23864126 PMC3834854

[6] Enke TN, Leventhal GE, Metzger M, Saavedra JT, Cordero OX 2018 Microscale ecology regulates particulate organic matter turnover in model marine microbial communities. Nat Commun 9:2743. doi:10.1038/s41467-018-05159-8. 30013041 PMC6048024

[7] Grossart H-P 2010 Ecological consequences of bacterioplankton lifestyles: changes in concepts are needed. Environ Microbiol Rep 2:706–714. doi:10.1111/j.1758-2229.2010.00179.x. 23766274

[8] Simon M, Grossart HP, Schweitzer B, Ploug H 2002 Microbial ecology of organic aggregates in aquatic ecosystems. Aquat Microb Ecol 28:175–211. doi:10.3354/ame028175.

[9] Kiørboe T 2001 Formation and fate of marine snow: small-scale processes with large-scale implications. Sci Mar 65:57–71. doi:10.3989/scimar.2001.65s257.

[10] Simon HM, Smith MW, Herfort L 2014 Metagenomic insights into particles and their associated microbiota in a coastal margin ecosystem. Front Microbiol 5:466. doi:10.3389/fmicb.2014.00466. 25250019 PMC4155809

[11] Crump BC, Armbrust EV, Baross JA 1999 Phylogenetic analysis of particle-attached and free-living bacterial communities in the Columbia River, its estuary, and the adjacent coastal ocean. Appl Environ Microbiol 65:3192–3204. doi:10.1128/AEM.65.7.3192-3204.1999. 10388721 PMC91474

[12] Rösel S, Grossart HP 2012 Contrasting dynamics in activity and community composition of free-living and particle-associated bacteria in spring. Aquat Microb Ecol 66:169–181. doi:10.3354/ame01568.

[13] Mestre M, Ruiz-González C, Logares R, Duarte CM, Gasol JM, Sala MM 2018 Sinking particles promote vertical connectivity in the ocean microbiome. Proc Natl Acad Sci U S 115:E6799–E6807. doi:10.1073/pnas.1802470115. PMC6055141 29967136

[14] Boeuf D, Edwards BR, Eppley JM, Hu SK, Poff KE, Romano AE, Caron DA, Karl DM, DeLong EF 2019 Biological composition and microbial dynamics of sinking particulate organic matter at abyssal depths in the oligotrophic open ocean. Proc Natl Acad Sci U S A 116:11824–11832. doi:10.1073/pnas.1903080116. 31127042 PMC6575173

[15] Trowbridge JH, Lentz SJ 2018 The bottom boundary layer. Annu Rev Mar Sci 10:397–420. doi:10.1146/annurev-marine-121916-063351. 29298139

[16] Bourgault D, Cyr F, Galbraith PS, Pelletier E 2012 Relative importance of pelagic and sediment respiration in causing hypoxia in a deep estuary. J Geophys Res 117:C08033. doi:10.1029/2012JC007902.

[17] Bertagnolli AD, Treusch AH, Mason OU, Stingl U, Vergin KL, Chan F, Beszteri B, Giovannoni SJ 2011 Bacterial diversity in the bottom boundary layer of the inner continental shelf of Oregon, USA. Aquat Microb Ecol 64:15–25. doi:10.3354/ame01504.

[18] Archambault P, Snelgrove PVR, Fisher JAD, Gagnon J-M, Garbary DJ, Harvey M, Kenchington EL, Lesage V, Levesque M, Lovejoy C, Mackas DL, McKindsey CW, Nelson JR, Pepin P, Piché L, Poulin M 2010 From sea to sea: Canada’s three oceans of biodiversity. PLoS One 5:e12182. doi:10.1371/journal.pone.0012182. 20824204 PMC2930843

[19] Thibodeau B, Lehmann MF, Kowarzyk J, Mucci A, Gélinas Y, Gilbert D, Maranger R, Alkhatib M 2010 Benthic nutrient fluxes along the Laurentian Channel: impacts on the N budget of the St. Lawrence marine system. Estuar Coast Shelf Sci 90:195–111. doi:10.1016/j.ecss.2010.08.015.

[20] Savenkoff C, Vézina AF, Packard TT, Silverberg N, Therriault J-C, Chen W, Bérubé C, Mucci A, Klein B, Mesplé F, Tremblay J-E, Legendre L, Wesson J, Ingram R 1996 Distributions of oxygen, carbon, and respiratory activity in the deep layer of the Gulf of St. Lawrence and their implications for the carbon cycle. Can J Fish Aquat Sci 53:2451–2465. doi:10.1139/f96-198.

[21] Vézina AF, Savenkoff C, Roy S, Klein B, Rivkin R, Therriault J-C, Legendre L 2000 Export of biogenic carbon and structure and dynamics of the pelagic food web in the Gulf of St. Lawrence part 2. Inverse analysis. Deep Sea Res Part 2 Top Stud Oceanogr 47:609–635. doi:10.1016/S0967-0645(99)00120-4.

[22] Bourgoin L-H, Tremblay L 2010 Bacterial reworking of terrigenous and marine organic matter in estuarine water columns and sediments. Geochim Cosmochim Acta 74:5593–5609. doi:10.1016/j.gca.2010.06.037.

[23] Tremblay L, Gagné J-P 2009 Organic matter distribution and reactivity in the waters of a large estuarine system. Mar Chem 116:1–12. doi:10.1016/j.marchem.2009.09.006.

[24] Ramachandran A, Walsh DA 2015 Investigation of XoxF methanol dehydrogenases reveals new methylotrophic bacteria in pelagic marine and freshwater ecosystems. FEMS Microbiol Ecol 91:fiv105. doi:10.1093/femsec/fiv105. 26324853

[25] Colatriano D, Ramachandran A, Yergeau E, Maranger R, Gélinas Y, Walsh DA 2015 Metaproteomics of aquatic microbial communities in a deep and stratified estuary. Proteomics 15:3566–3579. doi:10.1002/pmic.201500079. 26223443

[26] Jones SE, Lennon JT 2010 Dormancy contributes to the maintenance of microbial diversity. Proc Natl Acad Sci U S A 107:5881–5886. doi:10.1073/pnas.0912765107. 20231463 PMC2851880

[27] Hunt DE, Lin Y, Church MJ, Karl DM, Tringe SG, Izzo LK, Johnson ZI 2013 Relationship between abundance and specific activity of bacterioplankton in open ocean surface waters. Appl Environ Microbiol 79:177–184. doi:10.1128/AEM.02155-12. 23087033 PMC3536108

[28] Campbell BJ, Kirchman DL 2013 Bacterial diversity, community structure and potential growth rates along an estuarine salinity gradient. ISME J 7:210–220. doi:10.1038/ismej.2012.93. 22895159 PMC3526181

[29] Denef VJ, Fujimoto M, Berry MA, Schmidt ML 2016 Seasonal succession leads to habitat-dependent differentiation in ribosomal RNA:DNA ratios among freshwater lake bacteria. Front Microbiol 7:606. doi:10.3389/fmicb.2016.00606. 27199936 PMC4850342

[30] Lankiewicz TS, Cottrell MT, Kirchman DL 2016 Growth rates and rRNA content of four marine bacteria in pure cultures and in the Delaware Estuary. ISME J 10:823–832. doi:10.1038/ismej.2015.156. 26394004 PMC4796920

[31] Blazewicz SJ, Barnard RL, Daly RA, Firestone MK 2013 Evaluating rRNA as an indicator of microbial activity in environmental communities: limitations and uses. ISME J 7:2061–2068. doi:10.1038/ismej.2013.102. 23823491 PMC3806256

[32] Gilbert D, Sundby B, Gobeil C, Mucci A, Tremblay G-H 2005 A seventy-two-year record of diminishing deep-water oxygen in the St. Lawrence Estuary: the northwest Atlantic connection. Limnol Oceanogr 50:1654–1666. doi:10.4319/lo.2005.50.5.1654.

[33] Hébert M, Tremblay L 2017 Production and persistence of bacterial and labile organic matter at the hypoxic water-sediment interface of the St. Lawrence Estuary. Limnol Oceanogr 62:2154–2167. doi:10.1002/lno.10556.

[34] Shafiei M, Dunn KA, Boon E, MacDonald SM, Walsh DA, Gu H, Bielawski JP 2015 BioMiCo: a supervised Bayesian model for inference of microbial community structure. Microbiome 3:8. doi:10.1186/s40168-015-0073-x. 25774293 PMC4359585

[35] El-Swais H, Dunn KA, Bielawski JP, Li WKW, Walsh DA 2015 Seasonal assemblages and short-lived blooms in coastal north-west Atlantic Ocean bacterioplankton. Environ Microbiol 17:3642–3661. doi:10.1111/1462-2920.12629. 25244530

[36] Walsh DA, Lafontaine J, Grossart H-P 2013 On the eco-evolutionary relationships of fresh and salt water bacteria and the role of gene transfer in their adaptation, p 55–77. Lateral gene transfer in evolution. Springer, New York, NY.

[37] Painchaud J, Lefaivre D, Therriault J-C 1987 Box model analysis of bacterial fluxes in the St. Lawrence Estuary. Mar Ecol Prog Ser 41:241–252. doi:10.3354/meps041241.

[38] Painchaud J, Therriault J, Legendre L 1995 Assessment of salinity-related mortality of freshwater bacteria in the Saint Lawrence Estuary. Appl Environ Microbiol 61:205–208. doi:10.1128/AEM.61.1.205-208.1995. 16534903 PMC1388326

[39] Herlemann DP, Labrenz M, Jurgens KJU, Bertilsson S, Waniek JJ, Andersson AF 2011 Transitions in bacterial communities along the 2000 km salinity gradient of the Baltic Sea. ISME J 5:1571–1579. doi:10.1038/ismej.2011.41. 21472016 PMC3176514

[40] Buchan A, LeCleir GR, Gulvik CA, González JM 2014 Master recyclers: features and functions of bacteria associated with phytoplankton blooms. Nat Rev Microbiol 12:686–698. doi:10.1038/nrmicro3326. 25134618

[41] Teeling H, Fuchs BM, Becher D, Klockow C, Gardebrecht A, Bennke CM, Kassabgy M, Huang S, Mann AJ, Waldmann J, Weber M, Klindworth A, Otto A, Lange J, Bernhardt J, Reinsch C, Hecker M, Peplies J, Bockelmann FD, Callies U, Gerdts G, Wichels A, Wiltshire KH, Glockner FO, Schweder T, Amann R 2012 Substrate-controlled succession of marine bacterioplankton populations induced by a phytoplankton bloom. Science 336:608–611. doi:10.1126/science.1218344. 22556258

[42] Liu R, Wang L, Liu Q, Wang Z, Li Z, Fang J, Zhang L, Luo M 2018 Depth-resolved distribution of particle-attached and free-living bacterial communities in the water column of the New Britain Trench. Front Microbiol 9:625. doi:10.3389/fmicb.2018.00625. 29670597 PMC5893722

[43] Ottesen EA, Young CR, Eppley JM, Ryan JP, Chavez FP, Scholin CA, DeLong EF 2013 Pattern and synchrony of gene expression among sympatric marine microbial populations. Proc Natl Acad Sci U S A 110:E488–E497. doi:10.1073/pnas.1222099110. 23345438 PMC3568374

[44] Lage OM, Bondoso J 2014 *Planctomycetes* and macroalgae, a striking association. Front Microbiol 5:267. doi:10.3389/fmicb.2014.00267. 24917860 PMC4042473

[45] Orsi WD, Smith JM, Liu S, Liu Z, Sakamoto CM, Wilken S, Poirier C, Richards TA, Keeling PJ, Worden AZ, Santoro AE 2016 Diverse, uncultivated bacteria and archaea underlying the cycling of dissolved protein in the ocean. ISME J 10:2158–2173. doi:10.1038/ismej.2016.20. 26953597 PMC4989311

[46] Newton RJ, Jones SE, Eiler A, McMahon KD, Bertilsson S 2011 A guide to the natural history of freshwater lake bacteria. Microbiol Mol Biol Rev 75:14–49. doi:10.1128/MMBR.00028-10. 21372319 PMC3063352

[47] Mizuno CM, Rodriguez-Valera F, Ghai R 2015 Genomes of planktonic *Acidimicrobiales*: widening horizons for marine actinobacteria by metagenomics. mBio 6:e02083-14. doi:10.1128/mBio.02083-14. 25670777 PMC4337565

[48] Ghai R, Mizuno CM, Picazo A, Camacho A, Rodriguez-Valera F 2013 Metagenomics uncovers a new group of low GC and ultra-small marine. Sci Rep 3:1294–1298. doi:10.1038/srep02471. 23959135 PMC3747508

[49] Woebken D, Fuchs BM, Kuypers MMM, Amann R 2007 Potential interactions of particle-associated anammox bacteria with bacterial and archaeal partners in the Namibian upwelling system. Appl Environ Microbiol 73:4648–4657. doi:10.1128/AEM.02774-06. 17526789 PMC1932835

[50] Fuchsman CA, Kirkpatrick JB, Brazelton WJ, Murray JW, Staley JT 2011 Metabolic strategies of free-living and aggregate-associated bacterial communities inferred from biologic and chemical profiles in the Black Sea suboxic zone. FEMS Microbiol Ecol 78:586–603. doi:10.1111/j.1574-6941.2011.01189.x. 22066565

[51] Eloe EA, Shulse CN, Fadrosh DW, Williamson SJ, Allen EE, Bartlett DH 2011 Compositional differences in particle-associated and free-living microbial assemblages from an extreme deep-ocean environment. Environ Microbiol Rep 3:449–458. doi:10.1111/j.1758-2229.2010.00223.x. 23761307

[52] Jiang D-M, Kato C, Zhou X-W, Wu Z-H, Sato T, Li Y-Z 2010 Phylogeographic separation of marine and soil myxobacteria at high levels of classification. ISME J 4:1520–1530. doi:10.1038/ismej.2010.84. 20596070

[53] Acosta-González A, Rosselló-Móra R, Marqués S 2013 Characterization of the anaerobic microbial community in oil-polluted subtidal sediments: aromatic biodegradation potential after the Prestige oil spill. Environ Microbiol 15:77–92. doi:10.1111/j.1462-2920.2012.02782.x. 22626032

[54] Brinkhoff T, Fischer D, Vollmers J, Voget S, Beardsley C, Thole S, Mussmann M, Kunze B, Bler I-D, Daniel R, Simon M 2012 Biogeography and phylogenetic diversity of a cluster of exclusively marine myxobacteria. ISME J 6:1260–1272. doi:10.1038/ismej.2011.190. 22189493 PMC3358034

[55] Ghiglione J-F, Conan P, Pujo-Pay M 2009 Diversity of total and active free-living vs. particle-attached bacteria in the euphotic zone of the NW Mediterranean Sea. FEMS Microbiol Lett 299:9–21. doi:10.1111/j.1574-6968.2009.01694.x. 19686348

[56] Garneau M-È, Vincent WF, Terrado R, Lovejoy C 2009 Importance of particle-associated bacterial heterotrophy in a coastal Arctic ecosystem. J Mar Syst 75:185–197. doi:10.1016/j.jmarsys.2008.09.002.

[57] Zaikova E, Walsh DA, Stilwell CP, Mohn WW, Tortell PD, Hallam SJ 2010 Microbial community dynamics in a seasonally anoxic fjord: Saanich Inlet, British Columbia. Environ Microbiol 12:172–191. doi:10.1111/j.1462-2920.2009.02058.x. 19788414

[58] Stewart FJ, Ottesen EA, DeLong EF 2010 Development and quantitative analyses of a universal rRNA-subtraction protocol for microbial metatranscriptomics. ISME J 4:896–907. doi:10.1038/ismej.2010.18. 20220791

[59] Baker GC, Smith JJ, Cowan DA 2003 Review and re-analysis of domain-specific 16S primers. J Microbiol Methods 55:541–555. doi:10.1016/j.mimet.2003.08.009. 14607398

[60] Sanschagrin S, Yergeau E 2014 Next-generation sequencing of 16S ribosomal RNA gene amplicons. J Vis Exp 2014:e51709. doi:10.3791/51709. PMC4828026 25226019

[61] Schloss PD, Westcott SL, Ryabin T, Hall JR, Hartmann M, Hollister EB, Lesniewski RA, Oakley BB, Parks DH, Robinson CJ, Sahl JW, Stres B, Thallinger GG, Van Horn DJ, Weber CF 2009 Introducing mothur: open-source, platform-independent, community-supported software for describing and comparing microbial communities. Appl Environ Microbiol 75:7537–7541. doi:10.1128/AEM.01541-09. 19801464 PMC2786419

[62] Edgar RC, Haas BJ, Clemente JC, Quince C, Knight R 2011 UCHIME improves sensitivity and speed of chimera detection. Bioinformatics 27:2194–2200. doi:10.1093/bioinformatics/btr381. 21700674 PMC3150044

[63] Glöckner FO, Yilmaz P, Quast C, Gerken J, Beccati A, Ciuprina A, Bruns G, Yarza P, Peplies J, Westram R, Ludwig W 2017 25 years of serving the community with ribosomal RNA gene reference databases and tools. J Biotechnol 261:169–176. doi:10.1016/j.jbiotec.2017.06.1198. 28648396

[64] DeSantis TZ, Hugenholtz P, Larsen N, Rojas M, Brodie EL, Keller K, Huber T, Dalevi D, Hu P, Andersen GL 2006 Greengenes, a chimera-checked 16S rRNA gene database and workbench compatible with ARB. Appl Environ Microbiol 72:5069–5072. doi:10.1128/AEM.03006-05. 16820507 PMC1489311

[65] Schlitzer R 2015 Data analysis and visualization with Ocean Data View. CMOS Bull SCMO 43:9–13. hdl:10013/epic.45187.d001.

[66] Edgar RC 2004 MUSCLE: a multiple sequence alignment method with reduced time and space complexity. BMC Bioinformatics 5:113–119. doi:10.1186/1471-2105-5-113. 15318951 PMC517706

[67] Tamura K, Stecher G, Peterson D, Filipski A, Kumar S 2013 MEGA6: Molecular Evolutionary Genetics Analysis version 6.0. Mol Biol Evol 30:2725–2729. doi:10.1093/molbev/mst197. 24132122 PMC3840312

